# An ML-Based Approach to Reconstruct Heart Rate from PPG in Presence of Motion Artifacts

**DOI:** 10.3390/bios13070718

**Published:** 2023-07-07

**Authors:** José María Vicente-Samper, Christian Tamantini, Ernesto Ávila-Navarro, Miguel Ángel De La Casa-Lillo, Loredana Zollo, José María Sabater-Navarro, Francesca Cordella

**Affiliations:** 1 Neuroengineering Biomedical Group, Institute of Bioengineering, Miguel Hernández University of Elche, 03202 Elche, Spain; j.sabater@umh.es; 2 Unit of Advanced Robotics and Human-Centred Technologies, Department of Engineering, Università Campus Bio-Medico di Roma, 00128 Rome, Italyl.zollo@unicampus.it (L.Z.);; 3 Department of Materials Science, Optics and Electronic Technology, Miguel Hernández University of Elche, 03202 Elche, Spain; 4 Institute of Bioengineering, Miguel Hernández University of Elche, 03202 Elche, Spain

**Keywords:** heart rate, photopletismography, motion artifacts, artificial intelligence

## Abstract

The heart rate (HR) is a widely used clinical variable that provides important information on a physical user’s state. One of the most commonly used methods for ambulatory HR monitoring is photoplethysmography (PPG). The PPG signal retrieved from wearable devices positioned on the user’s wrist can be corrupted when the user is performing tasks involving the motion of the arms, wrist, and fingers. In these cases, the obtained HR is altered as well. This problem increases when trying to monitor people with autism spectrum disorder (ASD), who are very reluctant to use foreign bodies, notably hindering the adequate attachment of the device to the user. This work presents a machine learning approach to reconstruct the user’s HR signal using an own monitoring wristband especially developed for people with ASD. An experiment is carried out, with users performing different daily life activities in order to build a dataset with the measured signals from the monitoring wristband. From these data, an algorithm is applied to obtain a reliable HR value when these people are performing skill improvement activities where intensive wrist movement may corrupt the PPG.

## 1. Introduction

The heart rate (HR) is a widely used clinical variable that provides important information on the health status as it is also related to the individual’s blood pressure [1]. For example, a large continuous increase in blood pressure, which in turn leads to an increase in HR, may be a risk factor for primary hypertension or a symptom of coronary heart disease [2]. In addition, there are also studies showing that to maintain the resting HR at values substantially lower than the tachycardia threshold, traditionally defined as between 90 and 100 beats per minute (BPM) is advisable [3], despite the difficulty of defining the optimal HR for each individual.

On the other hand, the photoplethysmography (PPG) signal can be used to detect blood volume changes in microvascular tissue [4] and is often used in a non-invasive way to make measurements on the skin surface. This technology is integrated into a multitude of medical devices to measure oxygen saturation, blood pressure, or cardiac output, as well as to evaluate the autonomic nervous system activity and to detect peripheral vascular diseases [5]. The PPG measurement requires only a few optoelectronic components: a light source to illuminate the tissue (i.e., the user’s skin) and a photodetector that measures the small variations in light intensity associated with changes in blood volume. However, the interaction of light with biological tissue is very complex and includes optical processes of scattering, absorption, reflection, transmission, and fluorescence [6]. Therefore, it is not so easy to isolate the desired component in the PPG wave. In addition, the wavelength of the light source is also important in the interaction with the tissue [7]. Tissue is mainly composed of water that strongly absorbs light in the ultraviolet range and in the longer infrared wavelengths. However, there is a range in the absorption spectrum of water that allows the visible red light and the shorter wavelengths of the infrared range to pass more easily through the tissue, and thus blood volume can be measured at these wavelengths. For this reason, red or short infrared light sources are often used for PPG measurement [8]. Moreover, the wavelength also determines the depth at which the light penetrates the tissue, as the intensity of the optical radiation depends on it [9].

On the other hand, the autonomic nervous system is one of the main systems in the human body for maintaining the homeostasis [10]. The activity of the sympathetic system increases when a person is faced with changes or stressful situations. However, the parasympathetic activity increases during rest and relaxation [11]. The HR is controlled by modulations of both the sympathetic and parasympathetic systems [12], mainly influenced by a person’s circadian rhythms to maintain the homeostasis [13]. Consequently, in addition to the physiological conditions of the person and their circadian rhythms, the interaction with the environment and the psychological state of the person affect the balance of the autonomic nervous system, and this will be reflected in the user’s HR [14]. Therefore, HR provides information for predicting changes in the user’s state, especially for people who are so affected by stimuli and changes.

### Background

People with ASD suffer from sensory sensitivity problems that affect their behavior, attention span, and social interactions [15]. Therefore, measuring their HR can help to detect changes in their state that may be caused by the presence of a negative stimulus in the environment. In addition, people with ASD are very reluctant to use foreign bodies, so the use of invasive systems, such as electrodes, for the measurement of physiological signals, such as HR, is not recommended as these devices will be difficult for people with ASD to tolerate them. Thus, the use of PPG for the acquisition of the HR in people with ASD could be useful because it is less invasive in the person’s space and in terms of the simplicity of the measurement.

**Figure 1:**
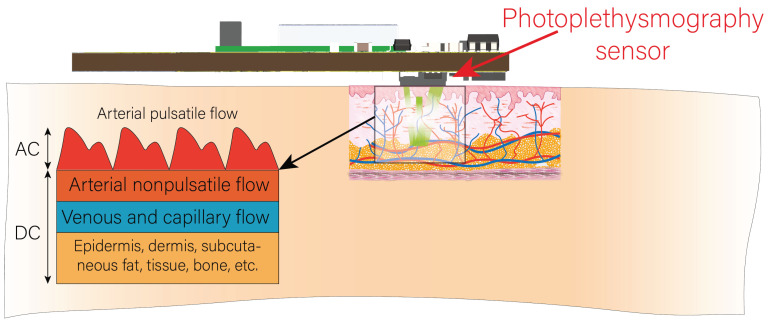
A recreation of the PPG sensor placement and measurement on the user. Includes a schematic of the main components that compose the PPG signal.

However, the PPG signal contains more information than is desired to extract, in addition to noise. The PPG signal has two main components, a pulsatile component attributed to cardiac changes in blood volume, and a slowly varying basal component attributed to respiration, sympathetic nervous system activity, and thermoregulation. The pulsatile component is often referred to as the “AC” component, due to its oscillatory behaviour-like AC power, and its fundamental frequency depends mainly on the HR, while the basal component is referred to as the “DC” component, due to its basal behaviour-like DC power, because it varies very slowly as its contributors have a lower frequency [16]. Figure 1 shows a picture of a PPG sensor placement on the user’s wrist and its working principles. It can be seen how the emitter sends the light pulses, and, after a series of optical phenomena, the light is reflected onto the photoreceptor. A schematic of the two components, described above, composing the PPG signal is also shown in Figure 1.

Due to the sensitivity of the PPG signal to the movements of the user’s arm, wrist, and fingers, when the user is performing tasks that involve constant movements of any of these parts, the PPG signal is corrupted and therefore the HR computed starting from the raw measurements significantly deviates from the real value [17]. The shown PPG sensor uses reflection detection. This principle is described by the Beer–Lambert law [18], from which the PPG measurement is defined in Equation (1), where $I_{i}$ and $I_{o}$ are the input and the reflected output intensities to the skin, respectively; ε represents the reflection/absorption coefficient of the different tissues; *c* is the concentration of the different tissues; and *d* represents the path length of the reflected light. Therefore, ε and *d* depend on the wavelength of the light, while *c* and *d* may vary in time if movements occur.

(1) $$I_{o}=I_{i}\cdot exp\left(-\sum_{j=1}^{n}\varepsilon_{j}\cdot c_{j}\left(t\right)\cdot d_{j}\left(t\right)\right)$$

**Figure 2:**
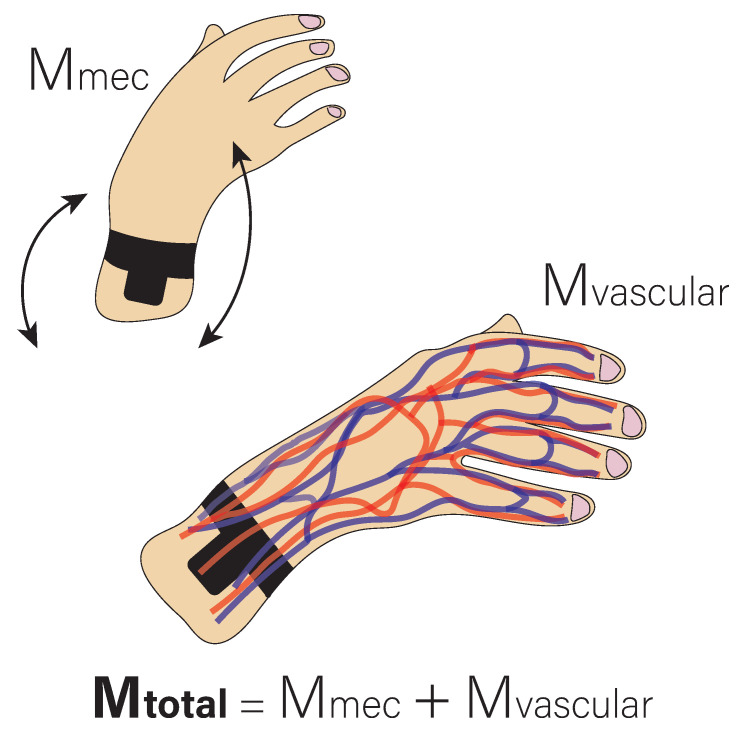
Simplified model of motion artifacts in the PPG signal.

According to Equation (1), there are thus two main sources of the motion artifacts: on the one hand, the displacement of the sensor relative to the skin (Mmec), which causes a change in the angle of incidence and the light path and which would correspond to the *d* term in Equation (1). On the other hand, the internal deformation and structural change of certain tissues due to finger movement (Mvascular), which produces an artifact in the signal and which would correspond to the c term. Figure 2 shows a simplified model of the previous point, where it is shown that the total motion artifacts are the sum of the artifacts caused by the displacement of the sensor and the artifacts caused by the changes in the microvascular tissues due to the movement of the fingers.

In the literature, several works present solutions for the removal of motion artifacts in the PPG signal. One of these solutions is explained in [19]. It proposes an algorithm for the removal of motion artifacts with the use of multiple PPG sensors with different wavelengths, one of them green, which will be in charge of measuring the user’s PPG signal, and another infrared, whose signal will be used as a motion reference. The proposed method performs a motion removal process based on the continuous wavelet transform (CWT), followed by HR estimation and signal reconstruction. The authors show results with low mean absolute error (MAE) in healthy users performing different activities with low physical intensity. Another work using different wavelengths is the one carried out in [20]. The authors propose the use of four sensors oriented in different directions that also have multiple PPG channels: one red, one green, and one infrared. In addition, a magneto-inertial measurement unit (M-IMU) is also incorporated to acquire the acceleration signal. When small or no motion is present, the HR is calculated from the PPG signal of the green sensor. In the case of motion, the PPG signal will be a mixture of the different sensors, which are oriented in different directions and measure different depths depending on the wavelength. An independent component analysis (ICA) algorithm is used to extract the pulsatile component of each sensor. Lastly, the FFT is applied to the component with the largest mean value to find the HR.

On the other hand, solutions based on the use of accelerometers to remove motion artifacts are also presented in the literature. In [21], a method that combines HR estimation with notch filtering is proposed. First, an adaptive least mean square algorithm (LMS) is applied to perform noise cancellation, which preliminarily reduces motion artifacts by using the acceleration signals of the three axes (x, y, and z) together with the PPG signal. Lastly, a notch filter is applied to subtract the frequencies associated with motion artifacts from the frequency response of the PPG signal, obtaining a separate noise-free signal. Similar work is carried out in [22]. The authors present a new algorithm based on spectral subtraction. For this purpose, the spectral component of the motion artifacts is estimated from the acceleration signals of the three axes (x, y, and z), and these components are removed from the frequency response of the PPG signal. This removes the peaks in the frequency response caused by the motion artifacts.

An alternative approach to the previously described techniques is the use of machine learning (ML) for HR detection from a PPG signal. ML can outperform adaptive filtering techniques due to its ability to learn autonomously, its ability to generalize to previously unseen data, its possibility to handle complex and non-linear features relationships, its flexibility in feature selection, and its scalability for large volumes of data. The work presented in [23] proposes the use of supervized learning using neural networks to face the problem without the need to use acceleration signals. Different peaks within the frequency spectrum of the raw PPG signal are selected, and a probability that each peak corresponds to the HR peak is assigned. Then, features are extracted and selected to train a three-layer neural network with 22 neurons in the hidden layer that will be in charge of estimating the HR value. Another work using ML techniques is [24]. It proposes the use of a four-layer deep neural network, two of which are convolutional neural network layers, together with two long short-term memory (LSTM) layers, followed by a dense output layer acting as a single-neuron regression layer, to predict the HR value from the PPG signal.

This paper is organized as follows. The Materials and Methods section describes the experimental setup and the proposed algorithm. Section 2.1 presents the used setup to obtain the HR by means of the PPG in people with ASD. In Section 2.2, a procedure to approach the identified problem is proposed. Section 2.3 explains the protocol for signal acquisition and processing to train the predictive models of the algorithm. Section 2.4 and Section 2.5 detail the training process of the classification and regression model, respectively. Section 2.6 describes the final architecture of the proposed algorithm. Section 3 shows the results obtained with several users in the validation stage of the algorithm. Lastly, in Section 4 and Section 5 the obtained results are discussed and the conclusions of the paper are outlined.

## 2. Materials and Methods

### 2.1. Experimental Setup

A monitoring device especially designed for people with autism spectrum disorder (ASD) is developed to measure physiological signals. The design of this device can be found in [25,26], and it is part of a more complex platform. This system is composed of different monitoring systems to acquire information from the environment and for the physiological user’s state in order to associate changes in the behavior of users with ASD with the stimuli of the environment. The developed device, which is a wristband, measures body temperature, motor activity by means of a 3-axis accelerometer and gyroscope, and PPG continuously on the user’s wrist. The integrated PPG sensor in the device (MAX86160 [27]) has two LEDs, one green and one infrared, which irradiate light pulses onto the skin of the user’s wrist. The reflected signal is used to detect changes in blood volume in the microvascular tissue, as explained above.

**Figure 3:**
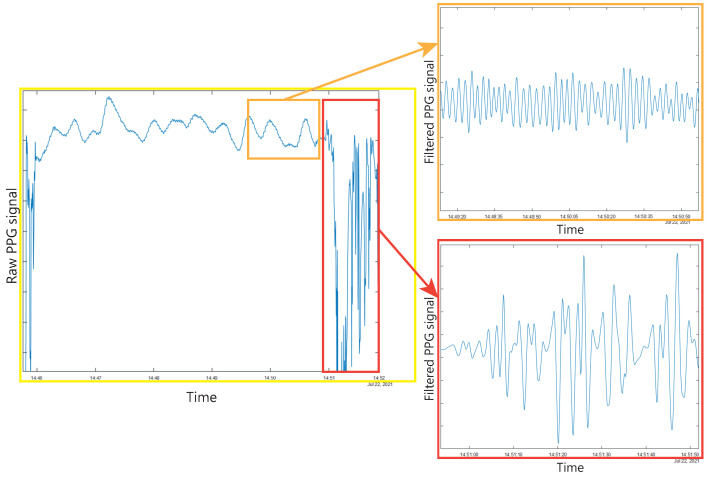
Plots resulting from the application of filtering to the raw PPG signal in periods with two different activities.

Due to the special characteristics of the users for which the physiological signal monitoring device has been developed, the device is attached to the user’s wrist in a soft manner since an excessively strong attachment may mean that the individual will not be able to tolerate the use of the system. This means that the signal intensity may be lower than the desired, and it will cause the signal to be even more sensitive to possible user movements. These conditions may mean that the obtained HR signal from the user during a monitoring period is partially corrupted or even almost entirely corrupted if the subject is uncooperative and the tasks performed are very intense. An example of this problem can be found in Figure 3, which shows a graph of the PPG signal measured on the wrist of a user during a test session. It can be seen how the shape of the signal changes throughout the session as the individual performs different activities during the session. If we try to apply common filtering (bandpass filter + median filter + low FIR filter) to the signal in different parts of the session, we will find different results for the different performed tasks. The orange rectangle corresponds to a time window where the user performs an activity with a low level of arm, wrist, and finger movement. It can be seen how, after applying the different filters to this part of the raw data, a clean signal is obtained, with a sinusoidal shape, and where the HR can be calculated with high confidence in the value. However, if we apply the same filters to a window corresponding to an activity where the user has performed constant movements of the arm, wrist, or fingers (red rectangle), the obtained signal is corrupted and, therefore, the HR calculation will probably have a result far from the real value and with very low confidence.

The works reviewed in the literature to solve this problem are focused on the removal of motion artifacts. Learning methods using techniques such as deep neural networks require a very high computational burden, especially if they are complex with the use of convolutional layers or LSTM [24]. The complexity of implementing these solutions in wearable devices to work in real-time is not an easy task.

Moreover, methods that try to subtract motion artifacts from the acceleration signals can be implemented in the system. However, these solutions do not usually take into account all the properties of the PPG measurement, or the micro-movements described above, including finger movements such as tapping or opening and closing the fist, which do not involve the movement of the arm. In the case of monitoring devices where the measurement of the PPG signal is performed on the wrist, the accelerometer, which is also on the wrist, is able to record the artifacts from the arm movements (Mmec) but cannot record the artifacts from the finger movements (Mvascular). Thus, the problem is that solutions that remove motion artifacts from the frequency spectrum of acceleration signals are not able to remove the component of tissue change caused by finger movements and even some wrist movements. For all of these reasons, a method that is able to cope with the presented problem is necessary.

### 2.2. Proposed Algorithm

**Figure 4:**
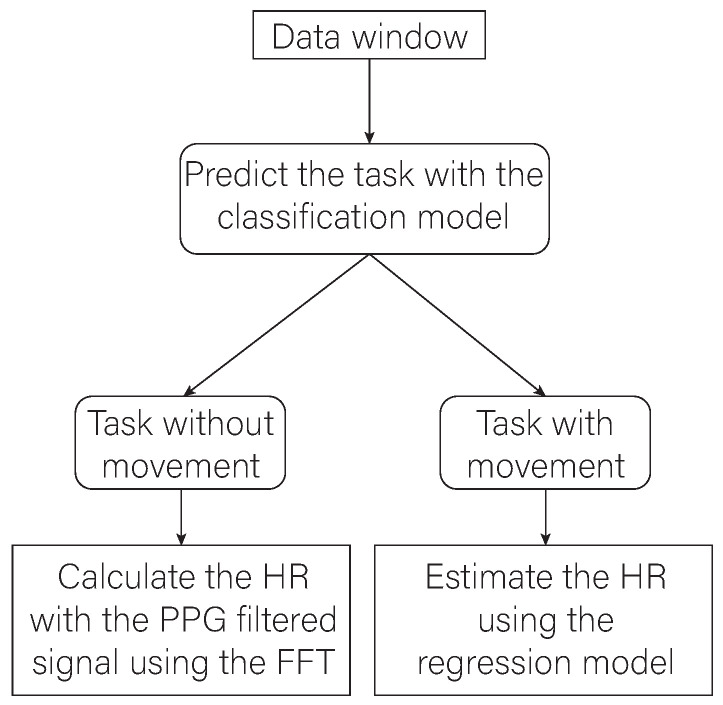
Diagram of the proposed algorithm.

The PPG processing pipeline proposed in this work is shown in Figure 4. It considers the use of all the available signals in the personal monitoring device (body temperature, acceleration, gyroscope, and PPG). The proposed approach is composed of two main parts: firstly, the approach generates and selects the appropriate features from the available signals to train a classification model that is in charge of detecting when the user is carrying out tasks involving arm and/or finger movements. It has been decided not to use the acceleration signals directly with thresholds to determine the instants in which there is motion, unlike some of the solutions analyzed in the literature, since the accelerometer signal placed on the wrist only captures arm movements and not finger movements, making it difficult to discriminate whether fingers are moving or not [20]. For the second part, the appropriate features are selected again, but in this case to train a regression model capable of estimating the HR. This second model will be used to calculate the HR in those instances in which the classification model determines that the user is performing a task in which the user is moving. Instead, the HR will be calculated from the filtered PPG signal when the classification model recognizes non-motion tasks performed by the user.

### 2.3. Signal Acquisition and Processing

In order to train the predictive models for the algorithm, having a sufficiently large and high-quality dataset is necessary so that the training of the models can be optimal and the results can be accurate enough to incorporate the work in the platform. For this purpose, an experiment has been carried out where different users have performed certain tasks while wearing the personal monitoring device presented before and also have installed the Zephyr BioHarness by Medtronic device [28]. This device consists of a chest strap and provides the user’s HR signal from the ECG measurement. This signal is used as the ground truth to evaluate HR in order to train the regression model in a supervized manner. BioHarness was selected since it is unobtrusive for the participants and has high accuracy in estimating HR. Numerous studies in the literature have already proven its validity at finding standard errors of measurement ≤5.9 bpm during high physical activity [29,30].

**Figure 5:**
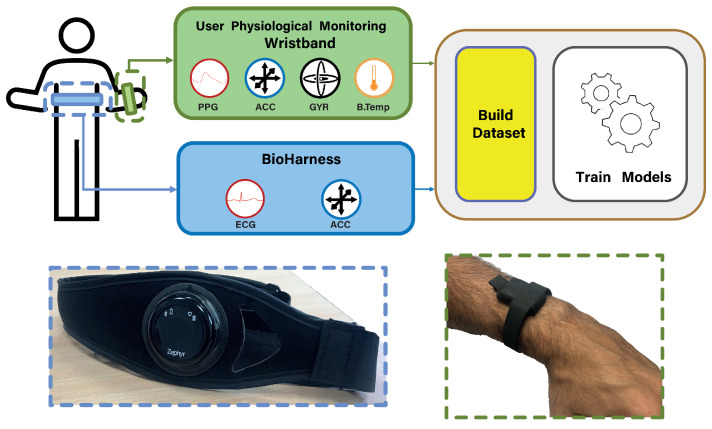
Workflow scheme for data recording using the BioHarness device in parallel with the physiological monitoring device.

For the acquisition of the signals, the diagram shown in Figure 5 has been followed. The user is equipped with the physiological signal monitoring device in his/her dominant hand and the BioHarness device correctly adjusted on the torso, in direct contact with the user skin. From the monitoring wristband, the four available variables (PPG, accelerometer, gyroscope, and body temperature) are obtained, from which the features that will make up the dataset entries are generated. The acquisition frequencies of the own device are 20 Hz for the PPG signal, 10 Hz for the accelerometer and gyroscope, and 0.1 Hz for the body temperature. The BioHarness collects the ECG and acceleration signals at acquisition frequencies of 250 Hz and 100 Hz, respectively. Given the raw ECG signal, the Pan–Tompkins algorithm is applied to accurately detect the QRS complex [31]. It consists of a sequence of filters that enhance the frequency content of the electrical activity of the heart and remove the background noise. Lastly, the algorithm includes the rectification of the signal in order to amplify the QRS complex to make it easy to identify. After this ECG pre-processing, the $IBI_{H}$ signal, also known as tachogram, is a signal defined as the temporal series of RR-peaks-intervals, i.e., the time between two consecutive R-peaks. Starting from the $IBI_{H}$ signal, it is possible to compute the instantaneous BioHarness HR ($HR_{BH}$), expressed as beats per min [bpm], as

(2) $$HR_{BH}\left(i\right)=\frac{60}{IBI_{H}\left(i\right)}$$

where *i* stands for the *i*-th detected R -peak. The signal extracted from the BioHarness accelerometer was used to synchronize data obtained from the two different devices during signal processing and dataset generation.

Lastly, the dataset will be composed of the features generated from the wristband signals and two extra parameters, one with the label of the real task that the user is developing at each instant, which will be used as output in the training of the classification model, and another one with the HR value of the BioHarness ($HR_{BH}$), which will be used as a label for the training of the regression model. The respective models will be trained from this dataset. Once both models have been trained, a second acquisition phase will be carried out where different users will perform the same experimental protocol to validate the developed approach with novel participants.

**Figure 6:**
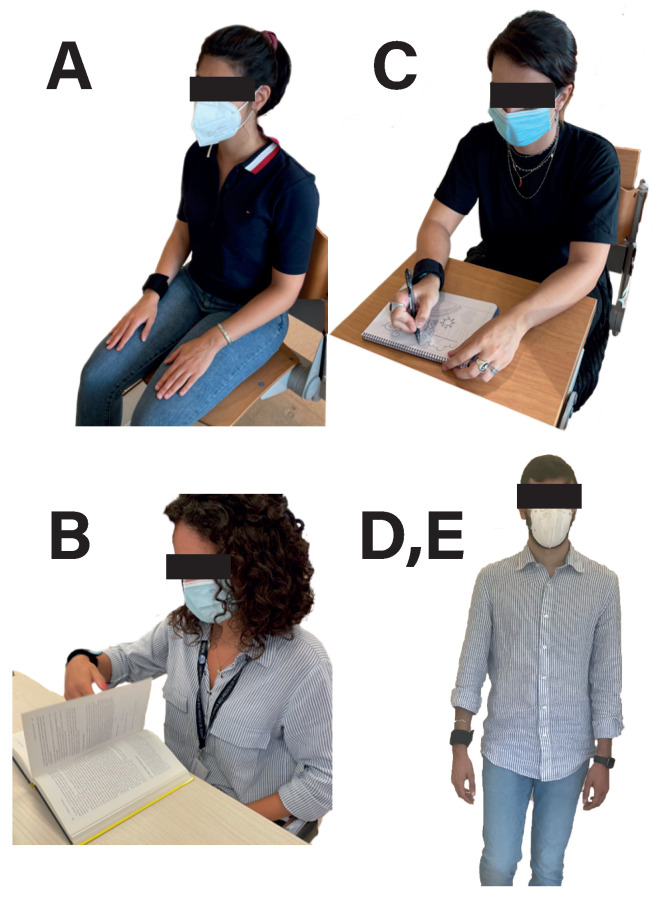
Pictures of users carrying out the different activities proposed for data recording ((**A**): Rest, (**B**): Read, (**C**): Write/Draw and (**D**,**E**): Walk slow,fast).

A protocol of 9 activities with a total duration of approximately 30 min was designed for data acquisition. The selected tasks for the experimentation consist of basic activities that are carried out during skills development sessions with people with ASD, and, therefore, the obtained knowledge can be extrapolated for these sessions. The study involved 10 different healthy users (5 women and 5 men) with a 27.2 ± 1.99 mean age. Table 1 shows the complete list of activities, and Figure 6 shows a picture of users carrying out the different activities. The tasks have been classified into 3 different groups depending on the type of movement and the intensity of the user’s movements. The group of activities in red corresponds to “resting activities”, where the user remains seated in a chair and relaxed, with the hand in a fixed position and trying not to make any movements. The green group corresponds to activities in which the user remains seated in the chair but performs light arm and finger movements more intensively. In task B, the user simulates reading a book by turning pages with the dominant hand periodically (approximately one page every 5 s), while in task C the users are asked to write and/or draw on paper in a natural fashion. The last group, in blue, corresponds to activities where the user is walking with their arms naturally swinging. In task D, the user has to walk at a slow pace, while in task E the user walks at an increased pace.

As a result of the experimentation, more than 5 h of signal recordings have been obtained. Before generating the features to build the dataset, some of the acquired signals were pre-processed to remove the noise. The signals from each axis of the gyroscope and the accelerometer are processed to remove the baseline from each one. From the pre-processed signals of each axis, the Euclidean norm or vector norm [32,33] of the gyroscope signal and the accelerometer signal is calculated using Equations (3) and (4), respectively.

(3) $$g=\sqrt{{g_{x}}^{2}+{g_{y}}^{2}+{g_{z}}^{2}}$$

(4) $$a=\sqrt{{a_{x}}^{2}+{a_{y}}^{2}+{a_{z}}^{2}}$$

For the PPG signal, a bandpass filter is applied to remove the high-frequency noise and artifacts of the low-frequency component (“DC”), due to temperature variations, changes in capillary density, etc. The frequency range for the bandpass filter is between 0.6 Hz and 3 Hz, where the fundamental frequency range of the HR is located. Then, an infinite impulse response (FIR) low-pass filter is also applied to smooth the signal and to remove the baseline [34]. Instead, the body temperature signal does not require any signal processing.

Once the signals have been processed, a collection of features is generated from them. The generated features are mainly statistical parameters: *Mean, median, standard deviation, mean absolute deviation, 25th percentile, 75th percentile, interquartile range, skewness, kurtosis, mode, truncated mean, geometric mean, harmonic mean, and variance*, as well as *the first and the second derivative in time* and *the fundamental frequency and the accumulated energy of the frequency response* (the last one defined as Equation (5), where FFT is the fast Fourier transform, conj, is the conjugate of a complex number and the $signal_{i}$, is the values array of the window *i*). The extraction of statistical features from time-varying signals makes it possible to reduce data complexity, synthesize information, increase robustness against noise, facilitate signal interpretation and analysis, and make the data compatible with machine learning algorithms [35]. Lastly, the features are normalized so that the mean of each group is 0 and the standard deviation is 1 by applying Equation (6), where $\bar{X}$ is the mean, σ is the standard deviation of the values array, and *x* is each one of the inputs in the values array.

(5) $$\mathrm{Energy}=\sum_{i=1}^{n}FFT\left(signal_{i}\right)\times conj\left(FFT\left(signal_{i}\right)\right)$$

(6) $$z=\frac{\left(x-\bar{X}\right)}{\sigma }$$

After aligning the data collected from the two devices exploiting the information obtained from the accelerometers, a 6-s time window [25] was used to calculate the input features from the wristband data and the average $HR_{BH}$ to be used as a label. In other words, regardless of the number of samples, i.e., different signal acquisition frequencies, features are calculated over a defined time window in the time domain (6 s). More specifically, only the features that have lastly been used for the training of the models belonging to the algorithm, 19 features, were extracted. As a result of this process, a dataset of 16,192 observations was obtained. Each of them consists of 63 different values from the generated features. In addition, the dataset incorporates the HR of the BioHarness as label 1 and the real task with 3 different possible classes as label 2, one for each group of activities. Therefore, the available dataset has a dimension of 16,192 entries and 65 columns. Before training the models, the data in the dataset are mixed and divided into two subsets: 70% for training and 30% for evaluation. The mixing of the data prevents overfitting, allowing the models to better generalize. Splitting the dataset allows the models’ performance to be evaluated on previously unseen data.

### 2.4. Training of an Activity Classification Model

The first of the models that makes up the proposed algorithm is in charge of classifying between each input into one of the three different task groups. For training, class 1 has been assigned to the group of resting tasks, class 2 to the group of tasks with specific finger movements, and class 3 to the group of tasks with user displacement. In addition, the “k-fold” cross-validation with a k equal to 5 has been used for the validation of the models during training.

**Figure 7:**
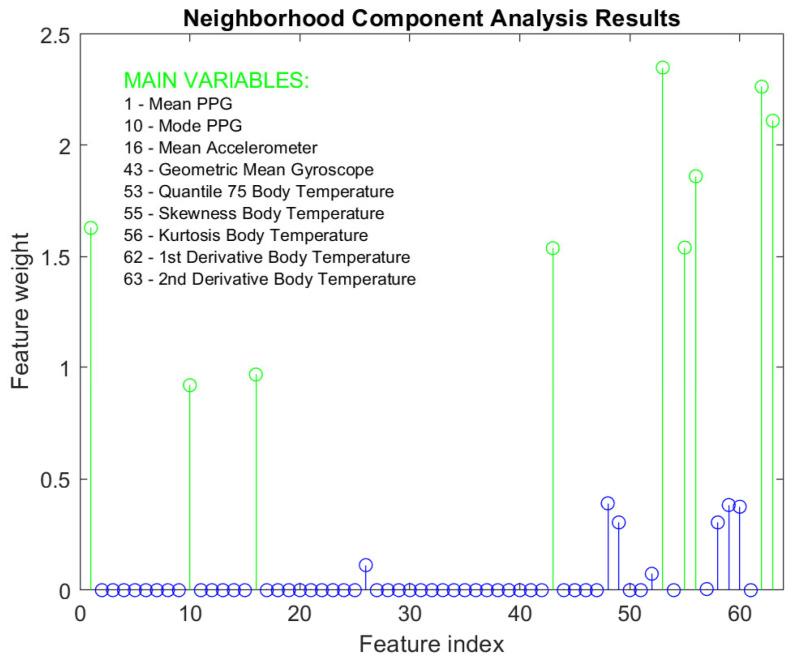
Result of applying the NCA algorithm to the dataset features with the weights of each feature.

Before training begins, a dimensionality-reduction algorithm is applied to establish which features in the dataset are the most important for the purpose of the model. The used algorithm is the neighborhood component analysis (NCA), a specific method for classification problems, which groups the features based on the results of the dimensionality reduction. It uses the variance of the components to estimate the number of clusters, similar to the k-means algorithm. NCA has been chosen since it offers several advantages over other similar methods in the literature. In particular, NCA allows for a targeted reduction in the dimensionality of the data, selecting the most informative features for activity identification and heart rate calculation. The algorithm takes into account local dependencies between data instances, allowing the most relevant features to be identified based on specific contexts or movement patterns. Another advantage of NCA is its supervized approach, which exploits the labeled information of the training data to guide the selection of relevant features. Compared to principal components analysis, NCA is more suitable as it is specifically designed to select the most relevant features for the task of interest, taking into account local dependencies between data instances. NCA, which directly identifies the most informative features by maximizing the separability of classes or target values, outperforms the recursive feature elimination approach [36], which recursively eliminates the least relevant features. Figure 7 shows the result of applying the algorithm to the training dataset. The weights of the different features according to their importance in classifying the activity can be seen. Those features that have a higher returned weight via the algorithm have been selected. This corresponds to the 9 attributes that are highlighted in green in the graph and listed together with their corresponding feature index.

Once the dimensionality-reduction algorithm has been applied, the training of the model is carried out by applying different algorithms. The *Matlab^®^ 2020b* Classification Learner tool was used to train the models. In particular, decision tree, K-nearest neighbors (KNN), support vector machine (SVM), bagged trees, and Nayve Bayes machine learning approaches have been tested. Such algorithms have been implemented since they are the most suitable to perform classification tasks dealing with physiological signals as input [37]. All these approaches were compared in order to find out which is the algorithm that best performs in terms of accuracy. Decision tree classifiers learn simple decision rules that can be inferred from observations: the deeper the tree, the more complex the decision rules that are generated. The principle behind the KNN method is to find a predefined number of training samples closest in distance to the new point and predict the label from these. SVM builds a decision boundary capable of classifying data belonging to different classes. Non-linear classification can be implemented by exploiting different kernel functions that can be applied to the input data. Bagged trees approaches extend the capabilities of the simple decision tree since they train an ensemble of models and return the best prediction as output. Lastly, naive Bayes (BYS) is one of the easiest ML algorithms to use to compute the class posterior probability to infer the prediction. It is based on the assumption that all the observations are independent. Table 2 shows the training result with the test dataset (30% data of the dataset) for different algorithms and compares the accuracy of the model trained with the 9 most important selected features according to the NCA with the model trained with the original 63 features of the dataset. It is observed that in general, the generated models with all the algorithms perform better when only the upper 9 features are used than when the full set is used. In addition, the algorithms are faster converging and are more optimized and lighter for implementing real-time inference. Among all the models, the bagged trees one has been selected, which has the highest accuracy (96.9%), followed by the KNN model (96.1%), but it is also slower than the selected model.

Bayesian optimization has been applied to the selected model to obtain the optimal hyperparameter values for the model. As a result of this optimization, the final model for task classification has the following characteristics:

$$\begin{array}{cc} \; & Algorithm=Bagged\;Trees\\ \; & Ensemble\;method=AdaBoost\\ \; & Number\;of\;learners=492\\ \; & Learning\;rate=0.34449\\ \; & Maximum\;number\;of\;splits=204\\ \; & Speed\;prediction=∼5800/s\\ \; & Training\;time=104.54\;s \end{array}$$

**Figure 8:**
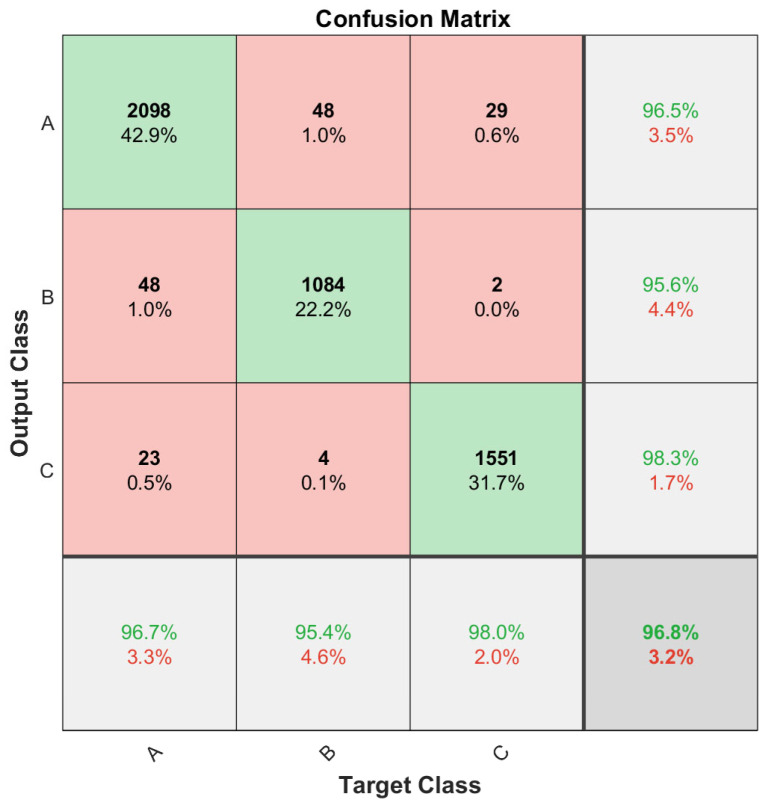
Confusion matrix resulting from the evaluation of the final classification model.

Figure 8 also shows the confusion matrix resulting from the evaluation of the model with the dataset reserved for this purpose. It can be seen that the model has an accuracy of 96.8% globally in predicting tasks against new data. For the group of tasks with user displacement, this percentage even increases to over 98% as the model identifies this type of situation more clearly. The few confusions that occur are mainly between resting and specific finger movement activities as the user maintains similar conditions in both tasks. However, it is a residual value as it only reaches 1% of the predicted values.

### 2.5. Training of a Heart Rate Estimation Model

**Figure 9:**
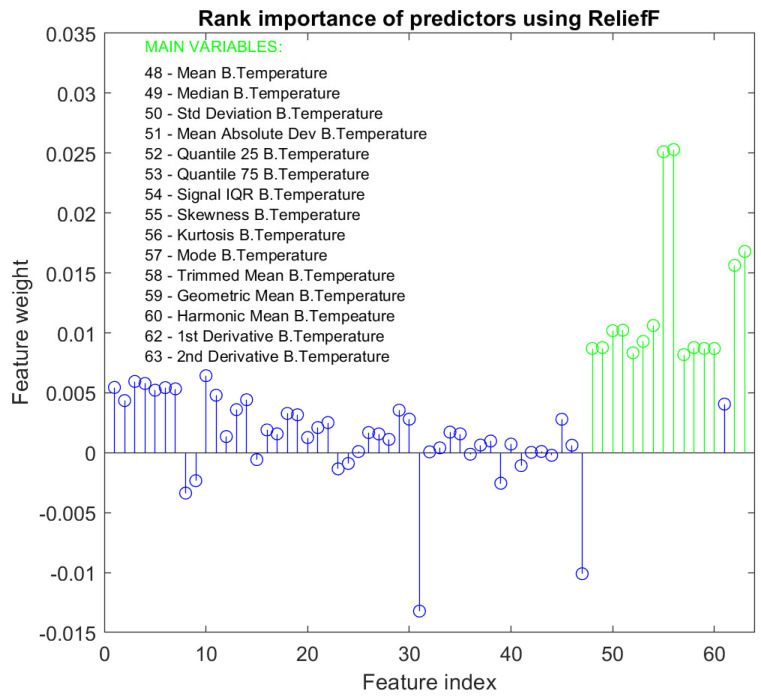
Result of applying the ReliefF algorithm to the features of the dataset with the weights of each feature.

The second model that forms the proposed algorithm consists of a regression model that estimates the HR value. For the training of this model, the HR value measured by the BioHarness is used as the output label. As with the training of the classification model, 5-fold cross-validation has been used to validate the models during training. Before the training, a dimensionality-reduction algorithm is again applied to establish which are the most important features of the dataset for the new target. In this case, the ReliefF algorithm [38] has been used. This algorithm penalizes predictors that assign different values to neighbors with the same response value and rewards predictors that assign different values to neighbors with different response values. Figure 9 shows the result of applying the algorithm to the training dataset. The weight of the different features can be observed according to the importance assigned by the ReliefF algorithm for the estimation of the HR. As in the classification model, the most relevant features have been selected. In this case, the algorithm returns a different scale than the one used in the classification model. The 15 selected features are highlighted in the graph in green and are also listed together with the corresponding feature index. It can be seen how the 15 selected features correspond to the body temperature variable. This seems logical since for the estimation of HR in situations where the PPG signal is corrupted, it does not provide valuable information to the model. The same occurs with the acceleration and gyroscope signals. These are signals that provide information to predict the task performed by the user but not to estimate the HR value.

Once the dimensionality-reduction algorithm has been applied, the training of the model is carried out using different algorithms to compare the results of the generated models. The *Matlab^®^ 2020b* Regression Learner tool was used to train the regression models. Decision tree, SVM, and bagged trees, already used in the classification task, can be also exploited to face HR regression. Moreover, linear regression and Gaussian process regression can be used in this case. The linear regression method represents the simplest manner to relate the predictors and a continuous predicted variable. On the other hand, such an approach can be too simple to face many realistic problems. Gaussian process regressors are models that exploit the probabilistic function to infer prediction. The root mean square error (RMSE ) is used to evaluate the performance of the implemented regressor since it provides a measure of the average error committed by the model with respect to reference values. It is sensitive to larger errors, penalizes outliers, and has an intuitive interpretation. RMSE can be computed as

(7) $$\mathrm{RMSE}=\sqrt{\frac{\sum_{i=1}^{n}{\left(HR_{BH}\left(i\right)-HR_{P}\left(i\right)\right)}^{2}}{n}}$$

where $HR_{P}\left(i\right)$ is the *i*-th predicted HR returned by the proposed approach and *n* is the total number of samples.

In order to determine if the number of acquired samples is sufficient to validate the proposed approach, a power analysis was conducted using the G*Power 3.1.9.7 software. A total of 860 samples were collected per participant during the experiments. The average measurements for $HR_{BR}$ and $HR_{P}$ were found to be 65.57±10.02 bpm and 67.09±11.04 bpm, respectively. The linear correlation between the two variables was calculated to be 0.25, indicating an effect size of 0.11. By setting a significance level of 0.05 and a desired statistical power of 0.80, the analysis determined that a minimum sample size of 449 samples is required. Given that experiments were conducted for a longer duration, the significance of the obtained results can be assured.

Table 3 displays training results on the test dataset for different algorithms. It compares the RMSE of models trained with 15 selected features from the ReliefF algorithm to those trained with 22 features using a lower threshold for feature selection. It is observed that adding more attributes to the models not only does not improve the training results but also slows down the models and makes the real-time integration of the system more difficult. Therefore, the use of the threshold to select the 15 most relevant features is justified. On the other hand, we have the comparative results between the different models. In addition, the 15 selected features are dependent on body temperature. This is because the PPG signal will be corrupted when it is necessary to estimate the HR value and the acceleration and gyroscope signals will change independently of the HR value. Among all of the models, two have been selected in this case: the one generated with the Gaussian process regression algorithm, with an RMSE of 5.07 BPM and whose performance in low HR ranges is better, and the one generated with the bagged trees algorithm, with an RMSE of 4.95 BPM and whose performance in high HR ranges is better. For the selected models, Bayesian optimization has been applied to obtain the optimal values of the hyperparameters for both models, as was done for the classification model. As a result of the optimization processes, the characteristics of both final models are presented:

- **Gaussian Process Regression Model**
  $$\begin{array}{cc} \; & Basis\;function=Constant\\ \; & Kernel\;function=IsotropicExponential\\ \; & Kernel\;scale=0.55637\\ \; & Standarize=Activado\\ \; & Signal\;standard\;deviation=14.331\\ \; & Sigma=69.2202\\ \; & Speed\;prediction=∼5300/s\\ \; & Training\;time=181.5\;s \end{array}$$
- **Bagged Trees Model**
  $$\begin{array}{cc} \; & Algorithm=Bagged\;Trees\\ \; & Ensemble\;method=Bag\\ \; & Number\;of\;learners=14\\ \; & Minimum\;leaf\;size=3\\ \; & Speed\;prediction=∼240,000/s\\ \; & Training\;time=1.26\;s \end{array}$$

As a result of the evaluation of the models with the test dataset, a total RMSE of 5.02 BPM has been obtained for the Gaussian Process Regression model. Regarding the partial RMSE obtained with this model, its value for task group 1, i.e., resting activities, is 5.42 BPM; for task group 2, i.e., activities with specific finger movements, is 4.48 BPM; and for the task group 3, i.e., activities with user displacement, is 4.84 BPM. The bagged trees model resulted in a total RMSE of 4.89 BPM, while the three task groups showed RMSE values of 5.20 BPM, 4.86 BPM, and 4.30 BPM, respectively.

### 2.6. Final Architecture of the Proposed Algorithm

**Figure 10:**
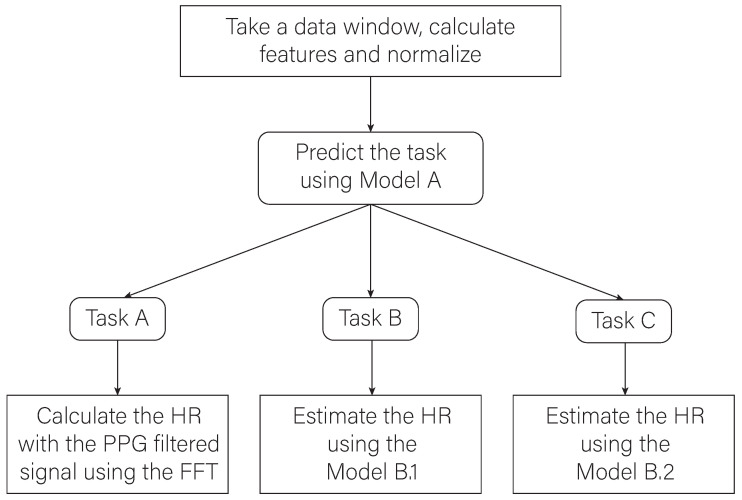
Graphical representation of the processes of the implemented algorithm.

Figure 10 shows a conceptual scheme where the final workflow of the proposed algorithm can be seen. Data collected in a time window of 6 s are processed to extract features and subsequently normalize them. A classifier is then trained to identify the task the participants are performing. Depending on the task that the user is performing and on the basis of the results obtained in the evaluation phase, three different ways to calculate the user’s HR are adopted. In particular, the calculation of the HR is carried out by applying the FFT of the filtered PPG signal if the classification model recognizes that the user is performing a task of group A, i.e., is at rest. The calculation of the HR will be carried out using one of the previously trained regression models if the result of the classification model is that the user is performing a task from groups B or C. In case the task belongs to group B, model B.1 will be used. It corresponds to the model generated with the Gaussian process regression algorithm, which has a better estimation performance for activities with lower FC value ranges. Instead, model B.2 will be used when the predicted task corresponds to group C. model B.2 corresponds to the model generated with the bagged trees algorithm, which performs better in activities where the HR values are higher.

## 3. Results

The performance of the algorithm proposed for the reconstruction of the HR signal acquired from different users is evaluated. For this purpose, the results of three representative participants in the second experimental phase are shown.

**Figure 11:**
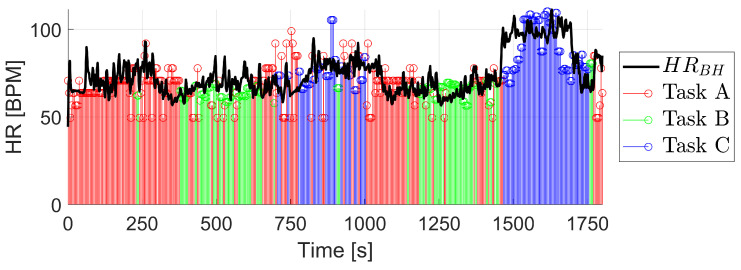
Result of the HR reconstruction in a training participant with high confidence in the BioHarness HR signal.

The first user showed high values of confidence in the BioHarness HR signal during the session, with an average of 95%. The result of the HR reconstruction is shown in Figure 11. The values in red in the graph correspond to those calculated by the method designated for the task A, i.e., using the filtered PPG signal and calculating its FFT. The points colored in green correspond to those calculated with the model designated for the task B, i.e., using model B.1. Lastly, the points colored in blue correspond to the values calculated with the model designated for task C, i.e., model B.2. In addition, the black signal corresponds to the HR signal measured by the BioHarness. It can be seen how the reconstructed signal follows the shape of the measured BioHarness signal during the session, although there are important differences at some points.

The performance of the algorithm is presented in Table 4. The algorithm is identifying correctly the task that is actually being performed with an 83.3% success rate. The algorithm has correctly identified task C almost every time, with a precision of 98% and a recall of 85%. Meanwhile, for tasks B and C the values decrease close to 80%, which indicates that these two groups of tasks are sometimes confused by the algorithm, presumably because the intensity of the activities is not very different from each other. Regarding the results of the calculated HR, the RMSEs are presented both globally and for each of the tasks. The global $RMSE_{global}$ is 12.20 BPM, while the error for task B, with the specific finger movements, drops below 8 BPM.

**Figure 12:**
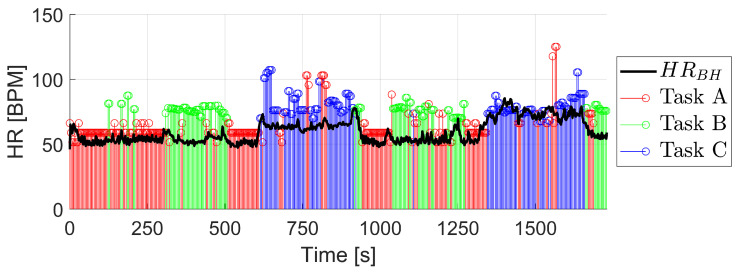
HR reconstruction on a new user with atypically low range in HR values.

The second user is characterized by an atypically low range of HR values, around 45 bpm at rest and a slow increase with intensified physical activity. Regarding the confidence in the BioHarness HR signal, the average is 79%. Figure 12 also shows the result of the reconstruction with the BioHarness HR signal. It can be seen how the reconstructed signal follows the shape of the BioHarness signal at all times. However, the values estimated by the algorithm are higher at all times than those of the BioHarness signal, except for higher intensity physical activity where the user’s HR values increase to more typical values. The cause of this is that the model has been trained with 10 users who had a typical range of the HR values and the model has learned from them during the training. The obtained results outlined that, in order to apply the developed approach on atypical participants, it is necessary to include in the database such information. It would certainly also improve the performance of the HR reconstruction for atypical subjects.

Table 5 presents the results of the algorithm for this user. The classification of the task has been performed with an 80% of success, again with almost perfect values when it comes to task C. The HR results are not significant as it was previously known that a new user was outside of the range of values of the training dataset. However, for task A, which is performed using the filtered PPG signal, the RMSE is 12.08 BPM, similar to the rest of the users as it does not depend on the training of the models.

**Figure 13:**
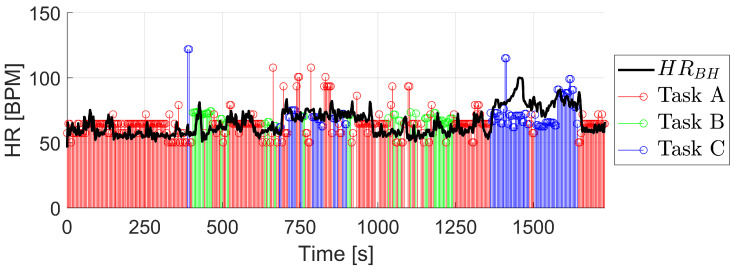
Result of the HR reconstruction on a new user with the typical range of HR values.

The last user is characterized by a typical HR value range and a mean BioHarness HR signal confidence of 85%. The result of the reconstruction together with the BioHarness HR signal from this session is shown in Figure 13. It can be seen how the reconstructed signal follows the shape of the BioHarness signal at all times and in this case with similar values between the two. In the last activity of task C, there is a difference in the range of values where the reconstructed signal starts the task with values below those measured by the BioHarness. This is due to the fact that the prediction model uses the features coming from the body temperature and it varies slower in time than the user’s HR. Therefore, the change in the user’s estimated HR by the algorithm is reflected with a delay relative to the measured signal by the BioHarness.

The results of the algorithm for this session are shown in Table 6. The classification of the task has been performed successfully 80.6% of the time, very similar to the rest of the sessions, with task C again being the best identified. Regarding the result of the calculated HR, the $RMSE_{total}$ of the session is 13.03 BPM, increasing for task C due to the previously analyzed reason.

## 4. Discussion

As can be seen from the results, the algorithm can reconstruct the user’s HR signal when the user is performing tasks with finger movements or displacements and the PPG signal is corrupted. The presented algorithm is able to predict the task performed by the user with high accuracy, and it allows for the estimation of the users’ HR when the PPG signal is corrupted. For this purpose, two regression models have been used whose inputs are features calculated from the physiological signals of the wristband. Moreover, the application of an automatic feature ranking algorithm to the collected dataset allows for computing of the feature weights that enable HR estimation. The application of the neighborhood component analysis revealed that body temperature is the most relevant feature. The average RMSE found in the validation phase is 12.23 BPM, while the average RMSE without using the proposed algorithm grows to 18.47 BPM. Furthermore, if the group of activities with the highest physical intensity (C) is evaluated, the average RMSE of the validation phase is 13.1 BPM, while the RMSE without using the algorithm is 22.47 BPM. The obtained RMSE allows for the reconstruction of those periods where the PPG signal is corrupted due to hand movement artifacts.The outcomes achieved through the implementation of the proposed machine learning pipeline for reconstructing HR from data extracted from the wristband exhibit promising results aligned with existing scientific literature. Notably, prior investigations have primarily focused on modeling distinct motion conditions to eliminate artifacts, encompassing periodic, random, and non-periodic continuous movements. Within these specific contexts, the reconstruction of HR has demonstrated the potential to reduce errors by up to 4.3 BPM [19]. Furthermore, when examining patients in a stationary position, an error margin of 5 BPM [24] has been obtained. In contrast, when concentrating on activities that entail significant movement, such as running, the methods proposed in the literature yielded errors in the range of 11.47 BPM [20,21,23]. It is crucial to highlight that the existing literature does not present any algorithms explicitly designed for HR estimation during routine daily activities, which could be of considerable interest in monitoring patients or children with ASD. Indeed, finger movements pose a notable challenge for HR reconstruction from PPG, and our study effectively demonstrates the efficacy of machine learning approaches in addressing this specific task.

From the presented results, it can also be extracted that if the model is trained with information from the user with whom the algorithm is going to be used, the error can be reduced since the model will learn information about the user’s behavior. This is especially important if the user’s range of HR values is atypical. For example, while the obtained average RMSE in the validation stage is 12.23 BPM, for a new user with outliers in the HR range the RMSE increases to 16.64 BPM. It may pose a setback when transferring the use of the algorithm to people with neurological disorders such as ASD as it may not be possible to train the model with the users’ information, provided by a device such as the BioHarness, as they do not usually tolerate this type of “invasive” system. However, solutions can be devised to correct this. For example, user information can be obtained from the signals of the monitoring wristband by analyzing them in detail to extract valid data where the PPG signal is not corrupted. Furthermore, from this information, it would be possible to work with an antagonistic generative network to generate synthetic data from the available data and thus increase the size of the dataset to train the model. In any case, using the proposed algorithm, even without being able to train it with particular information about the user, will allow the reconstruction of the HR signal in the sessions or parts of sessions where for some reason the PPG signal is corrupted, for example, due to high-intensity activities being carried out or because the user does not tolerate a correct fixation of the device on that day.

## 5. Conclusions

HR is a widely used clinical variable that provides important information on users’ physical status. One of the most commonly used methods for ambulatory HR monitoring is analyzing the PPG signal. Due to the sensitivity of the PPG signal to the movements of the user’s arm, wrist, and fingers, when a user is performing tasks that involve constant movements of any of these parts, the PPG signal is corrupted and therefore the HR obtained is also altered. This work presents an ML-based approach to reconstruct the user’s HR signal using a monitoring wristband, especially developed for people with ASD.

Experimental sessions were carried out to collect data from healthy participants and train the proposed pipeline. The promising results indicate that it is possible to recover an estimate of the HR even during activities involving finger movements.

The use of this algorithm together with the wristband improves the interaction between humans and the environment, e.g., children with ASD. In addition, it allows one to obtain their HR in situations where it is not possible to strongly fix the wristband or when they suffer a crisis and motion artifacts prevent them form obtaining a clean PPG signal.

Future works will be devoted to testing the proposed approach with ASD participants to test the effectiveness of the proposed ML pipeline to measure HR in the operative scenario.

## Figures and Tables

**Table 1:** List of performed activities during the data recording.

Activity	Time (min)
Rest (A)	5
Read (B)	3
Rest (A)	2
Walk slow (D)	5
Rest (A)	2
Write/Draw (C)	3
Rest (A)	2
Walk fast (E)	5
Rest (A)	2
**TOTAL**	∼30

**Table 2:** Results of training models for task classification with the 30% test dataset.

Algorithm	Accuracy (9 Features)	Accuracy (63 Features)
Decission Tree	94.3%	92.9%
KNN	96.1%	96.2%
SVM	91.3%	73.0%
**Ensemble: Bagged Trees**	**96.9%**	96.5%
Naive Bayes	76.8%	74.1%

**Table 3:** Results of training models for heart rate estimation with the 30% test dataset.

Algorithm	RMSE (15 Features)	RMSE (22 Features)
Decission Tree	5.08	6.93
**Gaussian Process Regression**	**5.07**	9.68
Support Vector Machine (SVM)	6.89	12.47
**Ensemble: Bagged Trees**	**4.95**	5.44
Linear Regression	16.4	18.43

**Table 4:** Results of a user session with high confidence of the BioHarness HR signal.

Task Classification				HR Calculation	
**Task**	**Precision**	**Recall**	**F1-Score**	**Task**	**RMSE (BPM)**
**1**	0.79	0.86	0.83	**1**	13.06
**2**	0.75	0.76	0.76	**2**	7.95
**3**	0.98	0.85	0.91	**3**	13.82
**Accuracy**		83.3%		**Total**	12.20

**Table 5:** Results of a new user with the range of HR values abnormally low.

Task Classification				HR Calculation	
**Task**	**Precision**	**Recall**	**F1-Score**	**Task**	**RMSE (BPM)**
**1**	0.71	0.86	0.78	**1**	12.08
**2**	0.73	0.70	0.71	**2**	22.91
**3**	0.98	0.81	0.89	**3**	16.49
**Accuracy**		80.0%		**Total**	16.64

**Table 6:** Results of a new user with typical range of HR values.

Task Classification				HR Calculation	
**Task**	**Precision**	**Recall**	**F1-Score**	**Task**	**RMSE (BPM)**
**1**	0.76	0.96	0.85	**1**	13.22
**2**	0.72	0.58	0.64	**2**	10.80
**3**	0.97	0.67	0.79	**3**	15.17
**Accuracy**		80.6%		**Total**	13.03

## Data Availability

The study did not report any data.
